# Vitamin D Deficient Older Adults Are More Prone to Have Metabolic Syndrome, but Not to a Greater Number of Metabolic Syndrome Parameters

**DOI:** 10.3390/nu12030748

**Published:** 2020-03-12

**Authors:** Henrique Pott-Junior, Carla Manuela Crispim Nascimento, Letícia Pimenta Costa-Guarisco, Grace Angelica de Oliveira Gomes, Karina Gramani-Say, Fabiana de Souza Orlandi, Aline Cristina Martins Gratão, Ariene Angelini dos Santos Orlandi, Sofia Cristina Iost Pavarini, Fernando Augusto Vasilceac, Marisa Silvana Zazzetta, Marcia Regina Cominetti

**Affiliations:** 1 Department of Medicine, Federal University of São Carlos (UFSCar), São Carlos 13565-905, Brazil; 2 Department of Gerontology, Federal University of São Carlos (UFSCar), São Carlos 13565-905, Brazil; carlamcnascimento@gmail.com (C.M.C.N.); lepcosta@ufscar.br (L.P.C.-G.); grace@ufscar.br (G.A.d.O.G.); gramanisay@ufscar.br (K.G.-S.); forlandi@ufscar.br (F.d.S.O.); alinegratao@ufscar.br (A.C.M.G.); sofia@ufscar.br (S.C.I.P.); fernando.vasilceac@ufscar.br (F.A.V.); marisam@ufscar.br (M.S.Z.); 3 Department of Nursing, Federal University of São Carlos (UFSCar), São Carlos 13565-905, Brazil; ariene@ufscar.br

**Keywords:** vitamin D, metabolic syndrome, inflammation, insulin resistance

## Abstract

This study investigated the relationship between metabolic parameters and low serum 25-hydroxyvitamin D (25(OH)D) levels in older adults (n = 265). They were assessed for anthropometrics and metabolic measurements, including 25(OH)D, insulin, glucose, total cholesterol (TC), high-density lipoprotein cholesterol (HDL-C), low-density lipoprotein cholesterol (LDL-C), triglycerides (TG) and other inflammatory markers. Vitamin D deficiency was defined as a 25(OH)D level below 50 nmol/L. Comparisons between groups were performed using Wilcoxon–Mann–Whitney or Pearson’s Chi-squared test. A multivariate adjusted Poisson regression was used to model the number of metabolic parameters as a function of a set of explanatory variables. Subjects with 25(OH)D deficiency were predominantly females and presented higher body weight, body mass index, waist circumference, triglycerides and Tumor Necrosis Factor-α (TNF-α), and higher insulin resistance. Metabolic syndrome was also more prevalent among 25(OH)D-deficient subjects. In those without metabolic syndrome, 25(OH)D deficiency was related only to obesity and higher insulin resistance. Female sex, hypertension, higher waist circumference and higher levels of hemoglobin A1C (%), HDL-C, and TG were significantly associated with an increased number of metabolic syndrome parameters after adjusting for covariates, but 25(OH)D was not. The fact that serum 25(OH)D concentration was inversely associated with metabolic syndrome and insulin resistance not only reaffirms the relevance to consider serum 25(OH)D concentration as an influencing factor for insulin resistance, but also the need to actively screen for hypovitaminosis D in all patients with this condition.

## 1. Introduction

Low serum 25-hydroxyvitamin D (25(OH)D) levels are a common finding in the elderly population and are not limited to bone health risks, but also beta cell dysfunction and increased insulin resistance, leading to metabolic diseases [1,2,3]. However, a straightforward association between low serum 25(OH)D levels and cardiovascular diseases is not yet clear, especially in the elderly population which typically has more than one associated risk factor.

According to the American College of Cardiology/American Heart Association, traditional cardiovascular risk factors include type 2 diabetes mellitus, high blood cholesterol, high triglycerides, high blood pressure, and overweight and obesity [4]. However, information on the usefulness of these traditional risk factors in older adults is limited, and it is likely that many of them lose their importance over time, while new ones appear.

More recently, many studies have reported an inverse association between serum 25(OH)D levels and cardiovascular risk. Low serum 25(OH)D concentrations (namely below 50 nmol/L) have been associated with increased risk of ischemic heart disease and myocardial infarction [5,6], heart failure [7,8,9], peripheral arterial disease [10], and cerebrovascular events [11,12]. However, the results of these studies may be limited by residual bias and reverse causality.

Therefore, although the relationship between metabolic parameters and cardiovascular risk is well known, the role of low serum 25(OH)D levels in this context has not yet been clarified. To this end, the authors have investigated the relationship between metabolic parameters and serum 25(OH)D levels in community-living older adults.

## 2. Materials and Methods

### 2.1. Study Population

We recruited a convenience sample of 265 adults aged 60 years or older from a community health center at São Carlos, São Paulo, Brazil. There were no excluding criteria for sampling. All subjects gave their written informed consent prior to their inclusion in this study. The study was conducted according to the guidelines laid down in the Declaration of Helsinki and all procedures involving human subjects were approved by the Federal University of São Carlos’s Research Ethics Committee (Number: 36167914.9.0000.5504).

#### General Obesity, Abdominal Obesity and Metabolic Syndrome

Body mass index (BMI) was calculated as weight (kg)/height (m)^2^ to evaluate the general obesity, and waist circumference (cm) as a measure of abdominal obesity. Metabolic syndrome was defined based on the National Cholesterol Education Program Adult Treatment Panel III guidelines (NCEP/ATPIII), updated by the American Heart Association and the National Heart, Lung, and Blood Institute in 2005 [13]. These criteria consider the presence of any three of the five following metabolic impairments: elevated waist circumference, elevated triglycerides, reduced HDL-C, hypertension and elevated fasting glucose. Insulin resistance was assessed by the homoeostasis model assessment of insulin resistance (HOMA-IR) = [Glucose (mg/dL) * Insulin (mU/L)]/405 [14].

### 2.2. Biochemical Parameters

In the morning after an overnight fast, venous blood was sampled for measurements of serum glucose, insulin, 25(OH)D, total cholesterol (TC), high-density lipoprotein cholesterol (HDL-C), low-density lipoprotein cholesterol (LDL-C), and triglycerides (TG), following routine biochemical laboratory protocols. Interleukin-10 (IL-10), Interleukin-1α (IL-1α), Interleukin-1β (IL-1β), Interleukin-6 (IL-6), Tumor Necrosis Factor-α (TNF-α), and Tumor Necrosis Factor-β (TNF-β, also known as lymphotoxin alpha) were assessed using a multiplex assay (Milliplex MAP Human Cytokine/Chemokine Panel; Millipore Corp., Burlington, MA, USA).

All serum 25(OH)D analyses were performed using LIAISON 25(OH)D Total Assay (DiaSorin, Saluggia, Italy) and, at the same, the laboratory. The assay is a fully automated competitive chemiluminescent immunoassay for the direct measurement of total 25(OH)D in human serum, EDTA plasma and lithium heparin plasma. The assay has a minimum detectable concentration of ≤5 nmol/L, a functional sensitivity of 17.5 nmol/L, and a precision (coefficient of variation) within-assay of ≤13% and inter-assay of ≤15% [9]. We used Endocrine Society’s Clinical Practice Guidelines to define vitamin D deficiency as 25(OH)D concentrations below 50 nmol/L [15]. Participants were also distributed into different groups according to their levels of 25(OH)D and to the presence of metabolic syndrome.

### 2.3. Statistical Analysis

Continuous data are presented as mean (standard deviation) or median [interquartile range] according to the Shapiro–Wilk test of normality. Categorical variables are presented as counts and percentages. Comparisons between groups were performed using the Wilcoxon–Mann–Whitney test for continuous variables, and Pearson’s Chi-squared test with Yates’ continuity correction for categorical variables. A stepwise multivariate linear regression was used to investigate the relationship between HOMA-IR and serum 25(OH)D concentrations. Covariates included in the final model were chosen based on minimization of the Akaike Information Criterion. A multivariate-adjusted Poisson regression was used to model the number of metabolic parameters as a function of those variables whose *p*-value did not exceed 0.1 in the crude regression analysis. Statistical significance was assessed at a two-sided *p* value < 0.05. All analyses were conducted using R version 3.5.3 (The R Foundation for Statistical Computing, Vienna, Austria) in R-Studio 1.1.463 (RStudio Inc., Boston, MA, USA).

## 3. Results

Subjects with 25(OH)D deficiency were predominantly females (69%, *p* = 0.03), and had an average age of 68 years (interquartile range [IQR], 64–75), as compared to those with adequate 25(OH)D levels. They also presented higher body weight (*p* < 0.001), mass index (BMI, *p* < 0.001) and waist circumference (*p* < 0.001), and higher insulin resistance (*p* < 0.001), levels of plasma TG (*p* = 0.02) and TNF-α (*p* = 0.03). The overall prevalence of metabolic syndrome was 44.2% (95% Confidence Interval [95% CI], 38.2%–50.1%) and was more prevalent among 25(OH)D deficient subjects (60.7% [95% CI, 50.3%–71.2%]; *p* < 0.001). There were no significant differences for other variables (see Table 1).

We further analyzed if there were significant differences conforming to the presence of metabolic syndrome. Table 2 shows that there were no differences in any variable within metabolic syndrome subjects, while in those without metabolic syndrome, subjects with 25(OH)D deficiency presented higher body weight, BMI, and waist circumference, and reduced insulin sensitivity. When comparing within metabolic syndrome subjects, serum levels of IL-10, IL-1α, and TNF-α revealed a trend towards higher levels in subjects with vitamin D deficiency.

The relationship between HOMA-IR and serum 25(OH)D concentration was further analyzed using a stepwise multivariate linear regression. Table 3 shows that 25% (Adjusted R^2^ = 0.23) of the variance in the final model was explained by female sex, body weight, waist circumference, and serum concentrations of triglycerides and 25(OH)D.

Considering that 25(OH)D levels could also have a direct relationship with an increased number of metabolic syndrome parameters, we performed a multivariate adjusted Poisson regression to model the number of metabolic syndrome parameters as a function of those variables whose *p*-value did not exceed 0.1 in the crude analysis. Table 4 shows, as expected, that hypertension, waist circumference, hemoglobin A1C (%), HDL-C, and TG continued being significantly associated with the number of metabolic syndrome parameters after adjusting for covariates, but this correction also showed that the association with 25(OH)D levels was due to interaction with other parameters. A noteworthy finding was that female sex was also independently associated with the number of metabolic syndrome parameters after adjusting for covariates.

## 4. Discussion

Our results showed that 25(OH)D-deficient subjects are more prone to having metabolic syndrome, but not to a greater number of metabolic syndrome parameters. This relationship seems to be more closely associated with fat distribution and insulin resistance.

As other metabolic parameters were also present in many of these subjects, we investigated whether metabolic and inflammatory markers would differ conforming to the presence of metabolic syndrome and 25(OH)D deficiency. To this end, our results showed that there was no statistically significant difference in any variable analyzed among metabolic syndrome subjects, while in those without metabolic syndrome, there was a significant difference towards a higher weight, BMI, and waist circumference, and higher insulin resistance in the vitamin-D-deficient group. Similar findings have been reported in the case of an inverse relationship between general obesity and serum 25(OH)D levels [16,17,18]. According to Wortsman et al. [19], this event could be explained by the “trapping” of cholecalciferol in the largest body fat pool of obese individuals. Alternatively, others have proposed that it may be associated with less exposure to UV radiation [20], lower vitamin intake and even a higher distribution volume for 25(OH)D [21]. Although relevant, this information does not explain why this finding was observed only in those without metabolic syndrome. For that, we assumed that this inverse relationship between 25(OH)D levels and obesity may be a milestone found at the beginning of the pathogenesis of metabolic syndrome, mainly due to its association with insulin resistance and related pathologies. In this regard, our results are also in line with those of many previous studies that demonstrated a correlation between HOMA-IR and serum 25(OH)D concentration.

On the basis of these results, we hypothesized that serum 25(OH)D concentration could have a direct relationship with the number of metabolic syndrome parameters. However, our results revealed that serum 25(OH)D concentration was not found to be independently associated with the number of metabolic syndrome parameters, whereas other traditional defining parameters of metabolic syndrome were. Similar results have been described previously, in which 25(OH)D levels were associated with metabolic syndrome in cross-sectional but not in longitudinal studies [22].

A noteworthy finding of our study was that female sex was also independently associated with the number of metabolic syndrome parameters after adjusting for covariates. This is not a novelty, but the significance of sex on the clinical expression and pathophysiology of this syndrome is still unknown [23]. Nevertheless, accumulating data suggest that there is a significant heterogeneity between men and women developing the metabolic syndrome, in large part due to hormonal regulation of fat distribution and attendant metabolic abnormalities [24]. In particular, it seems that this difference may be attributed to the lack of estrogen and its effects on glucose and lipid metabolism, as well as in fat distribution [25,26]. Thus, it seems reasonable that further studies consider sex differences in relation to the parameters of metabolic syndrome.

One limitation of this study is the cross-sectional design, which does not provide evidence of a temporal relationship between metabolic and inflammatory markers with 25(OH)D inadequacy. However, bias owing to variations between independent and dependent variables within the same individual was reduced. Another limitation was the lack of information on serum estradiol and 1,25-dihydroxyvitamin D concentration, the prevalence of osteoporosis, genetic analysis of single nucleotide polymorphisms of genes encoding vitamin D-binding protein and 25-hydroxylase, and on other potential confounders such as the use of supplements, clothing style and sunscreen use. Despite those, our results provide evidence of the relationship between metabolic syndrome parameters and inflammatory markers with 25(OH)D levels in a sample of community-living older adults.

Our study showed that serum 25(OH)D concentration is inversely associated with insulin resistance and metabolic syndrome, but not with inflammatory markers or the number of metabolic syndrome parameters. These findings not only reaffirm the relevance of considering serum 25(OH)D levels as a factor influencing insulin resistance, but also the need to actively screen for hypovitaminosis D in all patients with this condition.

## Figures and Tables

**Table 1:** Anthropometric parameters, inflammatory and metabolic profile in the population studied (n = 265).

Variable	25(OH)D ≥ 50 nmol/L (n = 181)	25(OH)D < 50 nmol/L (n = 84)	*p*
Age, years	68.00 [64.00, 74.00]	68.00 [64.00, 75.00]	0.8
Female sex	98 (54.1)	58 (69.0)	0.03
Current cigarette smoking	111 (61.3)	43 (51.2)	0.1
Anthropometric assessments			
Weight, kg	66.00 [58.00, 75.00]	73.00 [64.00, 82.00]	0.001
Height, m	1.56 (0.09)	1.56 (0.08)	0.7
BMI, kg/m^2^	27.41 [23.53, 30.86]	30.01 [26.31, 33.63]	<0.001
Waist circumference, cm	93.50 [83.00, 103.00]	100.00 [91.38, 107.62]	<0.001
Laboratory results			
Hemoglobin A1C, %	5.80 [5.40, 6.40]	5.80 [5.57, 6.73]	0.1
Total cholesterol, mg/dL	194.07 (43.67)	197.32 (37.05)	0.7
HDL-C, mg/dL	47.00 [40.00, 55.00]	45.50 [40.00, 52.25]	0.3
LDL-C, mg/dL	118.48 (34.31)	117.57 (33.73)	0.6
Triglycerides, mg/dL	125.00 [89.00, 172.00]	141.50 [99.75, 184.75]	0.02
HOMA-IR	1.16 [0.62, 2.25]	1.80 [1.12, 3.33]	<0.001
25(OH)D, nmol/L	62.50 [55.00, 77.50]	40.00 [32.50, 45.00]	<0.001
IL-10, pg/mL	0.60 [0.30, 1.70]	0.71 [0.40, 1.83]	0.2
IL-1α, pg/mL	1.40 [0.50, 7.70]	2.00 [0.50, 8.85]	0.2
IL-1β, pg/mL	1.30 [0.80, 3.10]	1.07 [0.69, 2.82]	0.2
IL-6, pg/mL	2.10 [1.40, 3.00]	1.80 [1.20, 3.71]	0.3
TNF-α, pg/mL	1.70 [0.78, 4.09]	2.25 [1.28, 4.49]	0.03
TNF-β, pg/mL	0.50 [0.20, 1.00]	0.50 [0.20, 1.41]	0.9
Metabolic Syndrome parameters			
Any three of the five criteria below	66 (36.5)	51 (60.7)	< 0.001
Hypertension ^a^	115 (63.5)	67 (79.8)	0.01
Abdominal obesity ^b^	89 (49.2)	60 (71.4)	0.001
Hyperglycemia ^c^	49 (27.1)	31 (36.9)	0.1
Dyslipidemia ^d^	59 (32.6)	37 (44.0)	0.09
Dyslipidemia ^e^	72 (39.8)	46 (54.8)	0.03

Continuous data are presented as mean (standard deviation) or median [interquartile range]. Categorical variables are presented as counts (percentages). ^a^ Blood pressure: >130 mmHg systolic or >85 mmHg diastolic or pharmacologic treatment. ^b^ Waist circumference > 102/88 for men/women. ^c^ Fasting glucose ≥100mg/dL or pharmacologic treatment. ^d^ Triglycerides (TG) ≥ 150 mg/dL or pharmacologic treatment. ^e^ High-density lipoprotein cholesterol (HDL-C) < 40/50 mg/dL for men/women or pharmacologic treatment.

**Table 2:** Characteristics of subjects with or without metabolic syndrome, according to the vitamin D level (n = 265).

Variable	Metabolic Syndrome			No Metabolic Syndrome		
	25(OH)D ≥ 50 nmol/L (n = 66)	25(OH)D < 50 nmol/L (n = 51)	*p*	25(OH)D ≥ 50 nmol/L (n = 115)	25(OH)D < 50 nmol/L (n = 33)	*p*
Age, years	68.00 [65.00, 72.00]	68.00 [64.00, 73.50]	0.7	68.00 [63.50, 76.00]	70.00 [63.00, 75.00]	0.8
Female sex	43 (65.2)	39 (76.5)	0.2	55 (47.8)	19 (57.6)	0.4
Current cigarette smoking	37 (56.1)	24 (47.1)	0.4	74 (64.3)	19 (57.6)	0.6
Anthropometric assessments						
Weight, kg	73.50 [66.25, 85.00]	77.00 [65.50, 85.00]	0.6	60.00 [54.00, 70.00]	66.00 [61.00, 75.00]	0.02
Height, m	1.56 (0.10)	1.56 (0.08)	0.9	1.57 (0.09)	1.57 (0.09)	0.9
BMI, kg/m^2^	30.68 [28.01, 33.26]	31.35 [28.61, 34.76]	0.3	24.88 [21.78, 28.23]	27.01 [24.17, 30.02]	0.03
Waist, cm	102.00 [95.00, 107.00]	104.00 [98.50, 109.00]	0.3	87.00 [78.00, 95.50]	94.50 [86.00, 102.00]	0.02
Laboratory results						
Hemoglobin A1C, %	6.40 [5.62, 6.80]	6.20 [5.65, 7.30]	0.8	5.60 [5.20, 6.20]	5.70 [5.30, 6.20]	0.4
Total cholesterol, mg/dL	197.70 (50.81)	195.86 (30.74)	1.0	191.99 (39.09)	199.58 (45.56)	0.7
HDL-C, mg/dL	45.00 [38.00, 49.00]	43.00 [38.00, 47.50]	0.8	50.00 [43.00, 58.00]	51.00 [45.00, 56.00]	0.7
LDL-C, mg/dL	118.61 (41.16)	112.55 (28.46)	0.6	118.40 (29.88)	125.33 (39.78)	0.7
Triglycerides, mg/dL	174.00 [141.00, 208.75]	166.00 [136.50, 240.50]	0.7	109.00 [83.00, 132.00]	102.00 [89.00, 134.00]	0.9
HOMA-IR	2.26 [1.33, 3.93]	2.41 [1.69, 4.34]	0.2	0.81 [0.54, 1.38]	1.07 [0.81, 1.35]	0.06
IL-10, pg/mL	0.45 [0.30, 1.46]	0.75 [0.35, 1.94]	0.09	0.72 [0.35, 1.73]	0.70 [0.40, 1.38]	0.7
IL-1α, pg/mL	0.85 [0.40, 2.75]	2.00 [0.55, 7.55]	0.06	1.70 [0.50, 9.85]	2.00 [0.50, 9.90]	0.7
IL-1β, pg/mL	1.20 [0.70, 2.62]	1.00 [0.80, 2.67]	0.7	1.60 [0.80, 3.95]	1.08 [0.60, 2.90]	0.1
IL-6, pg/mL	2.15 [1.52, 2.70]	1.80 [1.38, 3.87]	0.8	2.10 [1.30, 3.30]	1.68 [0.87, 2.80]	0.1
TNF-α, pg/mL	1.50 [0.69, 3.74]	2.27 [1.25, 4.21]	0.07	1.70 [0.80, 4.33]	2.09 [1.40, 4.71]	0.1
TNF-β, pg/mL	0.50 [0.20, 0.88]	0.90 [0.20, 1.58]	0.3	0.50 [0.24, 1.15]	0.34 [0.12, 1.00]	0.2

Continuous data are presented as mean (standard deviation) or median (interquartile range). Categorical variables are presented as counts (percentages).

**Table 3:** Results for the stepwise multivariate linear regression model (n = 265).

Dependent Variable	R^2^	Adjusted R^2^	*p*	
HOMA-IR	0.25	0.23	<0.001	
Independent variables	β Coefficient	Standard error	95% CI	*p*
Female sex	+0.46	0.27	−0.08–0.99	0.09
Weight, kg	+0.03	0.01	0.01–0.05	0.01
Waist, cm	+0.03	0.01	0.01–0.05	0.01
Triglycerides, mg/dL	+0.01	0.00	0.00–0.01	<0.001
25(OH)D, nmol/L	−0.02	0.01	−0.03–0.00	0.03

95% CI: 95% Confidence Interval.

**Table 4:** Crude and multivariate adjusted Poisson regression for modeling the number of metabolic syndrome parameters (n = 156).

Variable	Crude Analysis		Adjusted Analysis	
	PR (95% CI)	*p*	aPR (95% CI)	*p*
Sex				
Male	Reference	-	Reference	-
Female	1.41 (1.20–1.67)	<0.001	1.37 (1.13–1.65)	0.001
Hypertension ^1^	2.16 (1.76–2.67)	<0.001	1.75 (1.41–2.19)	<0.001
BMI, kg/m^2^	1.04 (1.03–1.06)	<0.001	1.00 (0.98–1.02)	0.7
Waist, cm	1.02 (1.02–1.03)	<0.001	1.01 (1.00–1.02)	0.007
Hemoglobin A1C, %	1.08 (1.04–1.11)	<0.001	1.04 (1.00–1.08)	0.04
Total cholesterol, mg/dL	1.00 (1.00–1.00)	0.1	1.00 (1.00–1.00)	0.8
HDL-C, mg/dL	0.98 (0.98–0.99)	<0.001	0.99 (0.98–0.99)	<0.001
LDL-C, mg/dL	1.00 (1.00–1.00)	0.9		
Triglycerides, mg/dL	1.00 (1.00–1.00)	<0.001	1.00 (1.00–1.00)	<0.001
HOMA-IR	1.09 (1.07–1.12)	<0.001	1.01 (0.98–1.05)	0.4
25(OH)D, nmol/L	0.99 (0.99–1.00)	<0.001	1.00 (1.00–1.01)	0.8
IL-10, pg/mL	0.99 (0.96–1.02)	0.6		
IL-1α, pg/mL	1.00 (1.00–1.01)	0.4		
IL-1β, pg/mL	0.99 (0.97–1.01)	0.4		
IL-6, pg/mL	1.00 (0.97–1.03)	0.8		
TNF-α, pg/mL	1.00 (0.99–1.01)	0.8		
TNF-β, pg/mL	1.00 (0.99–1.01)	0.9		

Abbreviations: PR, Prevalence Rate; aPR, Adjusted Prevalence Rate; 95% CI, 95% Confidence Interval. ^1^ Blood pressure: >130 mmHg systolic or >85 mmHg diastolic or pharmacologic treatment.
